# The complexities, coordination, culture and capacities that characterise the delivery of oncology services in the common areas of ambulatory settings

**DOI:** 10.1186/s12913-022-07593-3

**Published:** 2022-02-12

**Authors:** Bróna Nic Giolla Easpaig, Yvonne Tran, Teresa Winata, Klay Lamprell, Diana Fajardo Pulido, Gaston Arnolda, Geoff P. Delaney, Winston Liauw, Kylie Smith, Sandra Avery, Kim Rigg, Johanna Westbrook, Ian Olver, David Currow, Jonathan Karnon, Robyn L. Ward, Jeffrey Braithwaite

**Affiliations:** 1 Australian Institute of Health Innovation, Macquarie University, Level 6, 75 Talavera Road, North Ryde, NSW 2109 Australia; 2 Infant, Child and Adolescent Mental Health Services, Liverpool Hospital, Liverpool, NSW Australia; 3 South-Western Sydney Local Health District, Liverpool, NSW Australia; 4 South Western Sydney Clinical School, University of New South Wales, Liverpool, NSW Australia; 5 Ingham Institute for Applied Medical Research, Liverpool, NSW Australia; 6 St. George Cancer Care Centre, St. George Hospital, Kogarah, NSW Australia; 7 St. George Hospital Clinical School, University of New South Wales, Sydney, NSW Australia; 8 Faculty of Health and Medical Sciences, University of Adelaide, Adelaide, SA Australia; 9 College of Medicine and Public Health, Flinders University, Adelaide, SA Australia; 10 Faculty of Health, University of Technology Sydney, Sydney, NSW Australia; 11 Prince of Wales Clinical School, University of New South Wales, Sydney, NSW Australia; 12 Faculty of Medicine and Health, The University of Sydney, Sydney, NSW Australia

**Keywords:** Multidisciplinary care, Cancer outpatient, Ambulatory care, Patient-centred, Ethnography, Qualitative

## Abstract

**Background:**

Relatively little is understood about real-world provision of oncology care in ambulatory outpatient clinics (OPCs). This study aimed to: 1) develop an understanding of behaviours and practices inherent in the delivery of cancer services in OPC common areas by characterising the organisation and implementation of this care; and 2) identify barriers to, and facilitators of, the delivery of this care in OPC common areas.

**Methods:**

A purpose-designed ethnographic study was employed in four public hospital OPCs. Informal field scoping activities were followed by in-situ observations, key informant interviews and document review. A view of OPCs as complex adaptive systems was used as a scaffold for the data collection and interpretation, with the intent of understanding ‘work as done’. Data were analysed using an adapted “Qualitative Rapid Appraisal, Rigorous Analysis” approach.

**Results:**

Field observations were conducted over 135 h, interviews over 6.5 h and documents were reviewed. Analysis found six themes. Staff working in OPCs see themselves as part of small local teams and as part of a broader multidisciplinary care team. Professional role boundaries could be unclear in practice, as duties expanded to meet demand or to stop patients “falling through the cracks.” Formal care processes in OPCs were supported by relationships, social capital and informal, but invaluable, institutional expertise. Features of the clinic layout, such as the proximity of departments, affected professional interactions. Staff were aware of inter- and intra-service communication difficulties and employed strategies to minimise negative impacts on patients. We found that complexity, coordination, culture and capacity underpin the themes that characterise this care provision.

**Conclusions:**

The study advances understanding of how multidisciplinary care is delivered in ambulatory settings and the factors which promote or inhibit effective care practice. Time pressures, communication challenges and competing priorities can pose barriers to care delivery. OPC care is facilitated by: self-organisation of participants; professional acumen; institutional knowledge; social ties and relationships between and within professional groups; and commitment to patient-centred care. An understanding of the realities of ‘work-as-done’ may help OPCs to sustain high-quality care in the face of escalating service demand.

## Background

Multidisciplinary care (MDC) is considered best practice in the delivery of services to cancer patients [1–4]. A range of terms exist in the literature to reflect the mechanisms and models associated with this care, such as multidisciplinary teams or multidisciplinary clinics, reflecting the specific organisation and structure of a service [1–4]. MDC, in this paper, refers to care overseen by an identifiable team of professionals responsible for diagnosis (e.g., pathologists, diagnostic radiologists and clinicians) and treatment of cancer and its impacts (e.g., medical, radiation and surgical oncologists, psycho-oncologists, physiotherapists, occupational therapists and palliative care specialists) [5]. Whole-of-person, patient-centred care is the goal of this approach, with patients acting as partners in decision-making about their care [5]. Input from a broad range of experts is essential as people with cancer have both medical and non-medical needs, such as psychosocial, informational, practical, circumstantial and other support needs over the patient journey [6–9]. While accepted as best practice, it should be noted that it is difficult to unequivocally demonstrate that MDC positively impacts survival outcomes [10–12], at least in part because of differing definitions of MDC and methodological difficulties [11].

Some understanding of MDC has been derived from the study of multidisciplinary team meetings (MDTMs) - formal mechanisms for diagnosis, care planning and management, usually organised around a tumour stream (e.g., lung cancer MDTM). This work reveals potential benefits, such as learning together, teamwork and promoting adherence to evidence-based guidelines [13–15], and also identifies the challenges in implementing this practice, such as time management and resource adequacy [16–18]. Quality enhancement strategies, such as the development of tools to assess MDTM performance, have derived from these studies [19].

We know comparatively little, however, about how aspects of MDC coordination and cooperation occurs as part of day-to-day care provision in ambulatory settings. Outpatient clinics (OPCs) are important sites for the provision of cancer care. Not all patient cases are discussed at MDTMs or return to formal discussion at an MDTM after initial diagnosis and treatment planning. OPCs offer an opportunity for clinicians to modify treatment plans and introduce new partners in the provision of MDC by, for example, identifying emergent or hitherto unrecognised needs and responding to them directly or by third party referral. Moreover, and unlike most MDTMs, these settings deliver consultation and treatment services with the patient, necessarily, present.

A high-level of coordination is needed to actualise MDC [20], which has implications for organisation and practice in OPCs. Coordination is an ongoing challenge across the patient journey [21, 22]. Prior research indicates there may be a lack of delineation of the specific roles that health professionals play in the team, there can be poor information flow between providers, and conflicting information may be provided to patients [20, 23, 24]. The professional responsible for the coordination of care can change in the course of care. Sub-optimal coordination can lead to poorer outcomes for patients and wastes the time of both patients and professionals [25]. The incidence of cancer is increasing [26], and health systems, and the professionals working within them, are faced with delivering the highest quality care possible within tight budgets. An understanding of the real-world provision of oncology outpatient services may help inform this effort. This study seeks to add to this understanding by examining care provision in the common areas of OPCs.

### Research framework and aims

Complexity science offers a valuable framework for examining oncology OPCs, conceptualising them as self-adapting, multi-faceted systems embedded within a complex mesh of relationships, including other health services. Such systems follow their own internalised rules (not necessarily those prescribed by top-down authorities) and respond to internal and external disruptions [27]. Agents who are interconnected through formalised organisational structures also self-organise through less formal networks (e.g., referral networks, collegial networks, friendships) to achieve shared goals or bridge gaps in formal systems of service delivery [28]. As interconnected networks of agents interact and respond to environmental demands, they not only generate fluid social structures but also communicate, shape ideas and produce rules by which to practice, which may or may not over time become formalised into policy [29, 30]. Thus, it is vital to go beyond “work-as-imagined” (as depicted in formalised work prescriptions and official role delineations) to engage with how work is actually accomplished, “work-as-done” [31]. This study seeks to shed light on the latter. The aims of the research were to:

- develop an understanding of behaviours and practices inherent in the delivery of cancer services in OPC common areas by characterising the organisation and implementation of care; and
- identify general barriers to, and facilitators of, the provision of care in OPC common areas.

## Methods

### Design

A focused, purpose-designed, multi-site, multi-method ethnography was undertaken [32, 33]. Ethnography was selected for its suitability for capturing data on complex adaptive social systems and gathering nuanced, behavioural examples of “work as done” [32, 33]. The following methods were used: the organisation of care and everyday practice were examined by observing professionals in OPC settings; the knowledge of key professionals was elicited via interview-based discussions about their work; and analytical and interpretative strategies were used to develop detailed accounts of MDC in these settings [34]. A critical realist philosophical position was taken [35–37], whereby the rendered ethnographic accounts may be contextually and structurally mediated, offering insight into complex phenomena [36, 37]. Consultation and scoping activities were used to prepare for key informant interviews, non-participant observations and document review. Early data collection activities informed the focus of later activities, cumulatively facilitating the development of the ethnographic account.

### Study settings and participants

Four cancer OPCs, situated within two metropolitan health districts in Sydney, Australia, participated. Collectively, the OPCs offer extensive consultation and outpatient treatment services. As shown in Table 1, there is variation in services offered, service size and capacity.

**Table 1:** Overview of services at study sites

Service	Outpatient occasions of service	Chemotherapy spaces (chairs and beds)	Assessment spaces	Radiation treatment	Care coordination	Palliative care consult	Allied Health	Oncology pharmacy	On-site MDTMs	Teaching hospital
1	9630 in 2016/17	10 spaces	2	✘	✓	✓	✓	✓	✓ (limited range)	✘
2	40,624 in 2016/17	10 spaces	2	2	✓	✓	✓	✓	✓ (limited range)	✓
3	93,853 in 2016/17	21 spaces	4	4	✓	✓	✓	✓	✓	✓
4	10,400 between Jul 2010 - Jun 2011	10 spaces	✘	✘	✓	✓	✓	✓	✘	✓

Source: Information obtained via plans, reports and observations

Observations were undertaken of a range of professionals at the study sites, including nursing, care coordination, allied health, medical, clinical research, administrative and management staff. Formal navigator interviews were undertaken with cancer nursing and care coordination staff. Malterud, Siersma and Guassora’s (2016) [38] model for generating ‘information power’ in qualitative studies guided the sample size, which took into account the study aim, specificity of the sample, the envisioned quality of the interview interaction, analysis strategy and established theory.

### Procedure

#### Preliminary research

Publicly-available local reports and cancer plans were analysed to generate an initial description of the cancer services at each hospital. These profiles were used to map key areas for data collection. Scoping discussions with managers and key staff were held to understand the study sites, and input was sought from oncology service management to ensure local relevance and the feasibility of the proposed methods.

#### Recruitment

Information about the research, including contact details and participant information sheets, was disseminated by email, posters, and via information sessions for target professional groups at each site. OPC management was consulted and access granted.

### Fieldwork

#### Non-participant observations

Unstructured observations of the delivery of care were undertaken in OPC areas associated with consultation, waiting and treatment, but not within consultation rooms or treatment bays. This included observing planned and ad hoc collaboration between professionals from differing disciplines during the care delivery process and informal discussions between researchers and professionals. Handwritten fieldnotes recorded observations and reflections. Observations were undertaken primarily by one researcher (BNGE) who is trained in ethnographic research practice, with some observations undertaken by two fellow team members: a senior research fellow (GA) and a research assistant (TW), both of whom were trained by BNGE to enhance data compatibility. This facilitated the sharing and discussion of unfolding understandings.

#### Key informant interviews

**Fig. 1:**
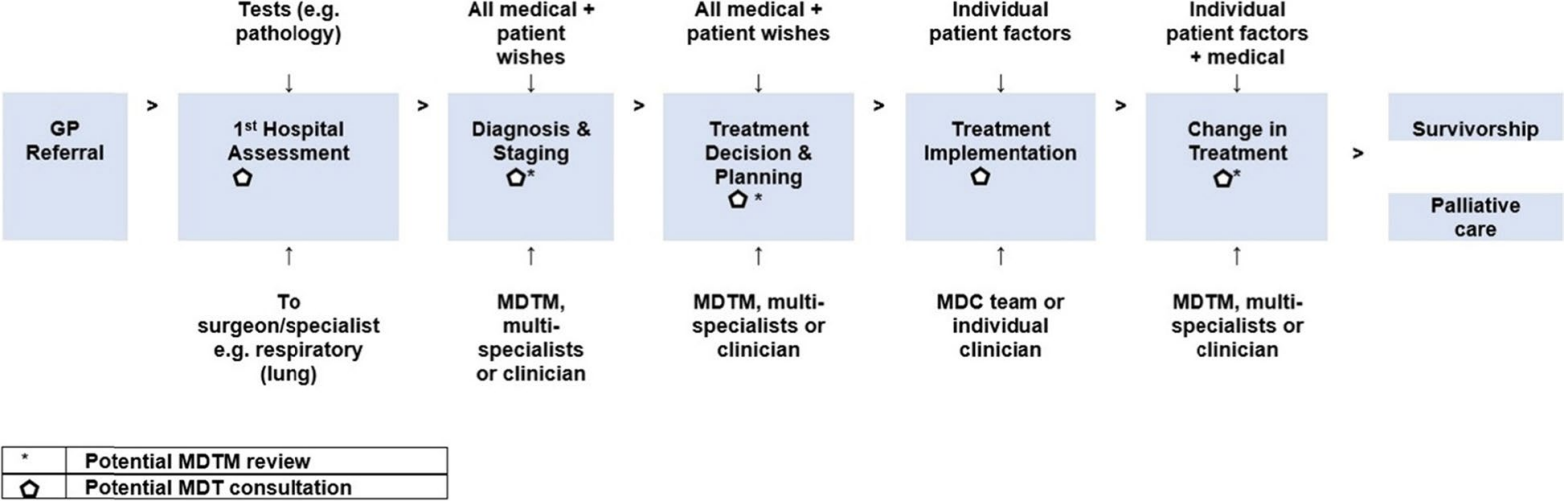
Care pathway map. Source: Author adaptation from Fennell et al., (2010) [40]

Formal, semi-structured interviews were undertaken with ‘navigators’, staff members with an overview of care pathways (a model or tool designed to guide optimal care planning, coordination and evidence-based cancer care) [39]. Interviews were oriented by care pathway maps derived from Fennell et al.’s (2010) [40], mapping of MDC in the patient journey (Fig. 1), which helped to gain an overview and contextualise OPC observations. Discussion concerned the organisation of practice and models of care, risk categories and complexity, as well as barriers and facilitators to practice. Interviews took an average of 30 min and were audio-recorded and transcribed. All interviews were undertaken by the same researcher (BNGE).

#### Document review

Relevant documents, identified during fieldwork, including unpublished policies and professional guidelines, were gathered and reviewed on an ongoing basis.

### Data management and analysis

Data were managed in accordance with ethical practice, as previously reported [41]. Reviewed documents were used to generate profiles of each site, to inform data collection and to contextualise observational and interview data. Ethnographic fieldnotes and interview transcripts were thematically analysed, oriented by an inductive approach [42]. The method of analysis for cross-site data was informed by the “Qualitative Rapid Appraisal, Rigorous Analysis” methodology developed by Phillips et al. (2014) [43]. First, all ‘like’ data (e.g., all interview data) was analysed for a site, followed by intra-site analysis of all data from one hospital OPC. Inter-site analysis then considered data across the sites to formulate salient themes. Subsequent inductive exploration revealed key underpinning characteristics of care, while synthesis allowed for the identification of factors which form barriers or facilitators to care.

A number of strategies appropriate to the methods were applied to safeguard quality, credibility and trustworthiness in the interpretation of the data. This involved verification and sampling of the coding strategy by experienced research team members and ongoing discussion of emerging interpretations within the team. Preliminary analysis was undertaken by one team member trained and experienced with this approach (BNGE), who developed a coding strategy which included coded data, interpretations and five suggested themes. Two experienced qualitative researchers (KL and DF-P) reviewed and verified the coding strategy to generate more nuanced understandings and facilitate revisions. One researcher (BNGE) further clustered the interpretations and consolidated the codes into six overarching themes, and assigned succinct phrases to the themes, in ongoing dialogue with the research team. Analysis was finalized in a team forum that did not alter the number of themes and factors identified, but which helped to develop greater conceptual definition between themes and refine the naming of the themes.

### Ethical approval

All methods were carried out in accordance with relevant guidelines and regulations. Informed consent was obtained from interviewees in written format. In adherence with the National Statement on Ethical Conduct in Human Research (2018) information about the observations was disseminated to target professional groups at each site by OPC authorities and via on-site briefings provided by members of the research team, prior to the data collection. As per the guideline, this information was provided in plain language, included study team contact details, instructions on how to opt-out of the observation and details about the secure management and use of data. Informational posters advised of the study and how to opt-out at any point up until the observation was completed. Written permission to observe was granted by authorities responsible for OPCs. Approval for the study was granted by a Local Health District Human Research Ethics Committee (no. 18/207).

## Results

The research was conducted over a 9-month period with observations undertaken in chemotherapy treatment areas, waiting rooms, consultation and other multipurpose areas (*n* = 135 h approximately). These common (non-private) areas are primarily serviced by nursing staff but can act as a hub for many different professional groups. Ad hoc, unsolicited discussions with professionals occurred naturally during this fieldwork. Care coordinators, tumour-specific specialist nurses, cancer nurse specialists and senior clinical staff participated in formal navigator interviews (n = 13), totalling six and a half hours. Notably, many of these key informants had worked across more than one of the hospitals, and in various roles such as multi-tumour or tumour-specialised care coordination and oncology nursing.

### Analysis: multidisciplinary care in real-world OPC practice

Analysis of these data resulted in the identification of six themes which captured OPC work as done: the care delivery team; professional identity and scope of practice; institutional expertise and capital; design and use of space; communication and flow of information; and negotiating patient-centred care.

#### Theme 1. Care delivery team

Working as part of a team was regarded as valuable, with staff members describing a shared respect and being in “awe” of their colleagues (key informant (KI)). The concept of “team” was at times taken to refer to a local professional disciplinary grouping (e.g., “chemotherapy nursing”) or to a cluster of different professionals working in a specific location.

The idea of flexible team membership was often discussed in relation to MDTMs. In response to issues that arose, staff members self-organised into teams comprising clinical and non-clinical professionals needed to best address the issue presented.

Administrative staff and other non-clinical staff, such as interpreters, frequently support care delivery, acting as communicators between nursing staff and doctors and in some cases even making efforts to advocate on behalf of patients. (Fieldnote p47)

In discussions, “team” was quite a fluid concept, especially where “we” and “they” (or equivalent) was used to identify oneself and others. “We” was not only employed to identify acts by the professional area of expertise (e.g.*, “We* educate them …” KI9), but was also used to direct cross-disciplinary collaboration:

The doctor was sent for, due to issues arising during pre-chemotherapy checks. The doctor comes to the nursing station and immediately begins speaking: “Right, what *we’ll* do is… [tasks for the nursing staff to undertake].” (Fieldnote p92)

These terms also highlighted distinctions between professional groups or groups with specific roles: “*we’re* a very close team” (KI2). “They” terms were often heard in situations where boundaries and responsibilities were negotiated, such as when a senior nurse discussed a patient with colleagues: “he [patient] is not supposed to be there—*they* put him here because they needed the space. Phone *them*.” (Fieldnote p280).

Notably, the representation of “team” was carefully enunciated in patient-facing interactions (discussion with or in front of the patient). “We” was used to extend the local team to encompass a broader multidisciplinary expertise (e.g., “here *we* have a psychologist, who is great” KI12), and to present a united approach:

Oncology nurse: “*We* were a bit worried about you” (referring to a concern of the doctor and the impetus for the change in care). (Fieldnote p256)

In patient-facing interactions “we” appeared to aid a sense of continuity across professionals and offer reassurance. Staff members attempted to shield patients from conflicting information or concerns about the continuity of care. Where there was potential for discord in this united approach, this was cautiously explored, often involving gentle probing for further information:

Where a patient complains about an ongoing side effect of treatment and felt it was not being taken seriously, the oncology nurse looks concerned and responds: “Did you tell the doctor? What did he say?” The oncology nurse confirms that the date of their next appointment with the doctor is soon and guides the patient on how to raise this issue with them again. (Fieldnote p174)

#### Theme 2. Professional identity and scope of practice

Professional identity, scope of practice and how individuals negotiate these issues with their colleagues emerged as salient for understanding MDC. Interviewees’ descriptions of their roles, their sense of identity, the associated professional values, as well as the passion they had, was expressed in the comment: “I love what I do, love the patients that I work with” (KI11). The application of professional expertise was observed even in the rare departure from the united approach often presented to patients:

The oncology nurse takes the decision not to administer chemotherapy and the patient voices their dissatisfaction. The nurse responds “you’ll need to go to [hospital], this is not safe anymore. I’ll talk to [doctor]. We are not comfortable to give you this.” (Fieldnote p98)

The respective roles and duties of differing professional groups tended to be cited in the context of potential conflicts. On occasion, a lack of mutual understanding of roles was noted.

While professional remit and duties may have been clear in theory, the scope and boundaries became less so in practice. The desire to prevent patients from “falling through the cracks” (KI13) appeared to contribute to staff members taking on duties which they did not believe was in their job description. This included where other services were overloaded, as illustrated: “I mean it should go totally to them [referring to a service outside of the hospital]. It doesn’t work that way though, it hasn’t for a long time because their staffing is so poor.” (KI5). In many cases the roles of staff members and teams appeared to adapt in response to the capacities of others and the demands made of them:

In response to the needs of the clinic, flexibility in clinicians’ tasks and role was observed. This appeared to be commonplace and expected. Further, staff were observed to go beyond their roles to facilitate care delivery, often becoming involved in other areas such as pharmacy, or even helping with parking issues. (Fieldnote p65)

This adaptability of roles and scope of practice illustrates the potential gap between work-as-imagined and work-as-done in the ambulatory setting.

#### Theme 3. Institutional expertise and social capital

In addition to professional expertise, a complementary form of “institutional expertise” was observed. Comprising organisational, cultural and social knowledge, this was a valuable resource, often deployed to navigate difficult situations:

Senior nursing staff who had worked in the district or the hospital for a number of years were often called upon where there was uncertainty or an issue. Their memory, history and experience were drawn upon to help navigate challenges. For example, where a patient might need to be admitted, the experienced staff would source information about the status of beds on wards and identify which specific staff members were occupying gatekeeping positions at that time. This was followed by negotiation and sometimes compromise with the relevant stakeholders/parties. (Fieldnote p122)

Because of professional and departmental interdependence, ‘bridges’, or staff who were familiar with or who moved between different areas of strategic importance, relayed information between the settings. These staff members (often senior or managing clinicians) were relied upon to relay status updates and real-time information, which informed near-term planning. As noted:

An experienced oncology nurse worked between the OPC and the ward. They were able to report on how busy other departments were, if there were any issues and the whereabouts/availability of key staff members who might be needed. This information was then factored into decision-making around securing staffing levels and patient transitions between areas. (Fieldnote p220)

Social capital (derived from status and position within social networks) formed another resource for front-line practice. This proved beneficial for navigating cross-disciplinary and interdepartmental issues and was most readily seen where senior nurses were approached by junior colleagues for help. For example:

The oncology nurse reported to the senior nurse that they had been waiting quite a long time for a surgical consultant to visit the patient and could not proceed until this happened. The senior nurse then phoned the consultant, and this request was framed within a humorous exchange, with the senior nurse declaring that the consultant owed the nursing staff coffee at the end of the call. (Fieldnote p256)

Relationships were recognised as vital in MDC practice, with staff members from across disciplinary areas investing in forming and sustaining interpersonal dynamics. At times, this simply involved an explicit commitment to cooperation, such as when a consultant confirmed to a senior nurse: “If he [patient] wants to see me again, page me and I will come down.” (Fieldnote p234).

These efforts could also be seen in the management of potentially tense situations, for example where a doctor was not satisfied with the way in which a nursing colleague had completed a form:

Oncology nurse: “With the form, do we need to fill out another?”

Doctor: “Yes, *it’s the strictest thing,* so *we* have got to make sure it’s perfect. Maybe hand do it.”

Oncology nurse: “Thanks [nickname for doctor].” (Fieldnote p134)

Maintenance of relationships, judicious deployment of social capital and the employment of institutional expertise supported formal care processes, and appeared vital for mitigating and resolving issues.

#### Theme 4. Design and use of space

Multi-site research permitted exploration of services with different treatment capacity (e.g., chemotherapy chairs/beds ranged from 10 to 21), on-site services (e.g., radiation therapy), and location of services such as allied health and oncology pharmacy. Features in the design and layout of OPCs emerged as important contextual mediators of MDC.

Sole and multi-purpose treatment areas created different opportunities for MDC interactions. In one OPC, the space and resources were organised to reflect the steps involved in chemotherapy preparation and administration; over the course of the observations, interactions with inter-professional colleagues were less frequently observed in this setting. In contrast, another OPC with a similar number of chemotherapy treatment spaces contained overlapping functional areas (e.g., it is necessary to walk through the chemotherapy suite to reach allied health) and parts of this space were thoroughfares to the ward and offices. Consequently, this OPC served as a meeting point to share information, find staff members and discuss concerns.

In the two smaller OPCs with multipurpose spaces, it appeared easier for allied health, treating physicians and other professional disciplines to casually visit patients while they were attending the clinic. This led to introductions, informal chats and consultations being undertaken while patients were waiting for or undergoing treatment (Fieldnote p57). In reality, even where spaces were not obviously multipurpose, they often functioned as such. For example, nurses working at the station of the chemotherapy treatment area were observed to field questions about a wide range of coordination issues, such as explaining parking and giving directions.

Where space was limited, staff adapted and used available free areas, including corridors and waiting areas; during fieldwork it became apparent that attempts to map key locations for multidisciplinary interaction in OPCs was futile as it occurred wherever staff met. Some staff members recognised the difficulties of working within limited space: “it is just that the work environment is quite small so it can be difficult to see patients and to deal with things that are coming up and changing” (KI8). Staff also highlighted the ways in which they tended to anticipate each other and act proactively to ensure consistent workflow given the confines of a small space.

The physical co-location of services and professionals influenced access to various expertise and input, as well as workflow in the chemotherapy treatment area. This was best seen in the interaction between pharmacy and chemotherapy staff. In one OPC, nursing staff could “just pop your head through” (Fieldnote p101), for immediate, in-person discussion with the pharmacist; this was valuable where there were schedule changes or when less common drugs were involved.

While at first glance the potential immediate access to inter-professional colleagues could be highly beneficial, a distinction was made between staff members being “present” and being “available” (KI9). For example, a common source of delay occurred where input from a doctor was needed to advise on whether chemotherapy should proceed, but the physician was not immediately available (e.g., in a consultation with a patient). Sometimes nursing or administrative staff spent time trying to track down the doctor in person. There was a recognition that doctors were attempting to prioritise and manage their, often multi-location, workloads. As one oncology nurse put it “you don’t own their time” (KI9).

#### Theme 5. Communication and flow of information

Co-location of consultation, treatment and other service areas was viewed as aiding communication “because you have all those prompts that you wouldn’t normally have” (KI13). This enabled opportunistic conversations, which staff found useful in situations “where you just need a quick, ‘yep that’s fine’” (KI13). Pursuing ad hoc communication, facilitated by physical co-location, was carefully exercised, taking into account urgency and appropriateness. Staff members were attuned to particular nuances, such as catching the doctor “between patients” or only calling in when the “door was ajar” (KI11). Unwritten/informal rules shaped communication practices:

[Discussing the delay that occurs when input is needed from a doctor to progress chemotherapy]

KI10: “Yeah. I guess a pager, but we don’t page them.”

Interviewer: “Is that because it's easier just to try and grab their attention?”

KI10: “I’m not sure. I’ve just been told just find them. If they are not there, just wait. I guess if they're in a meeting or with a patient you would only page them if it was an emergency.”

Drawing on institutional knowledge, methods of communication were often individually tailored, for example: “no, she won’t check that for ages, email her, she’s quick on that” (KI6).

It was not possible to observe electronic communication, such as emails or the oncology information system firsthand in the fieldwork; nevertheless, as this was a frequent topic of conversation, these discussions are pertinent in this theme. The potential benefits of centralised and shared oncology information systems for MDC were recognised by staff members. As one professional put it: “the psychologist will have documented that [they’ve] seen the patient and different things that need to be noted around that and we can pop in and we can see that” (KI8). Conversely, electronic documentation was noted as not always being well-integrated in the workflow. As one staff member reported: “So you might go, ok everyone is actually okay, so I’m going to… I will quickly sneak in [to the electronic system] and do this and catch up with the documentation that’s required” (KI8).

Electronic documentation could also provide a sense of protection for staff by providing a record of events: “if something were to happen, I need to provide that evidence [in order] to say, ‘no hold on, this is the communication trail’” (KI3). However, electronic communication was viewed as cumbersome and unreliable at times, especially when information was needed immediately, as one staff member expressed: “I’d rather pick up the phone than e-mail” (KI5). Staff members did not always appear confident about whether the information was up to date, as indicated by staff double-checking the information verbally and cross-checking with colleagues whether this “sounded right” (Fieldnote p167).

Staff members were very aware of potential communication issues and took steps to mitigate the risk of possible pitfalls:

Following the deterioration of a patient, the oncology nurse involved in their care discussed their status with a senior nurse. The senior nurse asked the oncology nurse whether they might be available to switch shifts, so that they could be there when the consultant arrived, to handover and liaise the following day. (Fieldnote p72)

Points of transition in care appeared to be of particular concern, and this included transitions between services in the same district, as noted:

“it's okay to go, we'll treat the patient here and then treat them over there …but it's about continuity of care. And, again, I may know something and if, [that service] doesn't know [it, they] might potentially miss something.” (KI12)

Coherent with the patient-facing approach described earlier, staff were vigilant about the potential negative impacts that communication issues could have on patients, and attempted to carefully manage this:

“we've had to say, unless [it is clear the patient has been informed], we're not touching it […] I've said ‘hi my name is [name] I’m the [role] at [hospital]’ and it’s literally, ‘do I have cancer? Nobody told me. How come no one told me?’” (KI2)

Staff members invest time in attempting to untangle confusing and conflicting information from sources such as caregivers, interpreters, electronic systems and other staff members in order to clarify events and ensure continuity of care for the patient.

#### Theme 6. Negotiating patient-centred care

MDC occurred in many areas in the OPC, but nowhere was patient-centred care better exemplified, in the areas observed, than when the patient was in the chemotherapy suite:

The oncology nurse shows the patient in and prepares them for treatment, they conduct an assessment (e.g., weight), ask about any issues and follow up with ongoing concerns. Following discussion with a patient, the oncology nurse states: “yep, yep, still no good, let me just…”. The oncology nurse pages the dietician who arrives about 20 minutes later to speak to patient. (Fieldnote p287)

There was a “while you are here” (KI6) approach taken, as various professionals used this time for impromptu consultations. In some cases, it appeared opportune for several separate patient needs to be addressed when the patient attended the clinic. While there are alternative forums for engagement, this approach appeared to overcome barriers, including for patients for whom English language proficiency is a barrier:

“I must admit, if it is a post op patient that I haven't met, I often won't call them [beforehand]. I wait till they are on the ward, arrange an interpreter or see them in clinic when I know the interpreters are there because it's too difficult to have a three-way conversation.” (KI7)

Interaction between staff and patients was frequently typified by humour, empathy and familiarity, as illustrated in the interaction between an oncology nurse and a patient: “oh yes that’s right, and what was it, what is he called again, your fish?” [Discussion about a patient’s pet fish]. (Fieldnote p101).

Staff members negotiated a tension between the time taken to provide this care and competing demands on the clinic. As captured by one interviewee:

“It’s also the high patient load, it is really difficult. Sometimes you can see that a patient has been sitting in the waiting room for like 45 minutes. Then you bring them in and they wait another 30 minutes. Then you still can’t check the chemo [two oncology nurses need to review the information prior to chemotherapy administration] … and it sucks because you know they don’t want to be here, and they are having a [bad] day just from being here.” (KI10)

To this end, staff members within nursing teams sought to work together optimally, but were concerned about short-staffing, and worried about the implications if they took leave. In day-to-day practice staff members sought to shield patients from this tension and ensure the preservation of patient dignity:

Upon discovering that a patient had become confused and not taken a medication required in advance of chemotherapy treatment, the oncology nurse consulted the senior nurse, who organised it so that the patient could receive the drug intravenously (lengthening their time in the chair and impacting the schedule). While explaining the adaption to their care, the nursing staff minimised the issue and joked with the patient about being a “trouble-maker” who kept them on their toes, as after all, someone needed to. (Fieldnote p234)

### Synthesis, barriers and facilitators

The four Cs—complexity, coordination, culture and capacity—were identified as key characteristics of MDC in ambulatory care settings, acting as threads that bind the themes. From this synthesis, factors which serve as barriers or facilitators were derived.

#### Complexity

Staff variously navigate the complexity, or manoeuvre within it, in an attempt to make their way in organisational settings and coordinate their own and others’ activities. The delivery of MDC relies on coordinated and timely input from various professionals, each responsible for managing their workloads; misalignment of their respective priorities may present a barrier. Here, individual acumen is an asset, where team members make decisions about the ongoing allocation of their time, including assessing costs associated with participating in ad hoc collaboration and care. The self-organisation of staff, often across professional boundaries with shared superordinate goals, helps to tackle issues which require a multidisciplinary response. This was observed where administrative staff helped to coordinate between doctors, managers, pharmacists and chemotherapy nurses to secure appropriate approvals and obtain a newly-approved drug available via a compassionate access scheme. While cross-department collaboration was challenging at times, bridges linking these networks and the benefit of institutional knowledge helped staff to predict responses and informed forward planning.

#### Coordination

The complexities of cancer care pose coordination challenges for staff, patients and their caregivers. OPC observations offer a glimpse into the plethora of services and professionals involved at a time point in the patient journey. While many aspects of care coordination are anticipated and planned, we noted the emergence of issues at the point of care. Descriptions of the themes provide examples of how staff members responsively adapt (e.g., paging the dietician to consult with a patient while they are receiving chemotherapy [Fieldnote p287]), sometimes aided by co-location, prioritisation and flexibility in staff members’ time and roles, as well as the fluidity of team membership. There was an awareness among staff about the potential pitfalls in coordination within and between services, and staff deployed institutional knowledge and flexibility in their roles to anticipate and address gaps (e.g., covering additional aspects of care where the relevant service did not have the capacity [KI5]). There was a concerted effort to preserve continuity of care for patients, with staff shielding patients from failures of communication or coordination.

#### Culture

MDC necessitates interdependent input from individuals each with their own respective professional identities and values. An appreciation of a colleague’s expertise, time constraints (e.g., “we don’t own their time” [K9]) and boundaries can enable collaboration. However, even where this appreciation was evident, misaligned priorities or pressure could potentially strain these relationships, forming barriers to MDC delivery. Social ties across disciplines provided channels for information flow, promoted cooperation and helped to dissipate tension. Often the social capital and institutional expertise of senior staff was called upon, offering an opportunity for junior colleagues to learn about the informal social conventions employed to sustain harmony and navigate difficulties. Striving for patient-centred care was core to professional identity, and connectedly the impetus to go “above and beyond” (K7) to achieve this appeared deeply ingrained within the culture.

#### Capacity

A range of supports and resources are needed to facilitate practice in ambulatory settings. A key tool for staff is the electronic oncology information system, as this has capacity to centralise pertinent information, refer to colleagues (e.g., allied health) and to communicate changes. However, the doubts staff expressed about the recency of information suggest this system is not perceived as optimal, and potentially may be a barrier. Much of the ad hoc collaboration arising as a result of the identification of needs during clinic visits was made possible by the availability, proximity and workload adaptations of relevant staff members. Sufficient staffing was cited as an issue, with some professionals concerned about letting down colleagues, or in some cases resulting in limits on services offered (e.g., it was not possible to open assessment spaces in OPCs without a certain staffing profile).

## Discussion

This study builds on the understanding of the provision of MDC in cancer services by developing an account of how, in practice, this care is delivered in ambulatory settings, mostly the chemotherapy consultation and treatment zones which were present in all four OPCs studied, and mostly delivered by nurses. Professionals endeavoured to provide holistic, patient-centred care, while simultaneously negotiating demands and pressure. Overwhelmingly, the main facilitator of the provision of quality care in these settings is the empathy of staff, their professional commitment to do their bit to optimise patient outcomes. The main barrier, resource constraint, acts directly to reduce care quality (e.g., through time pressure) and indirectly by creating tensions between staff and departments.

We captured nuanced, behavioural examples of “work as done” and developed a framework for understanding the four key issues that shape the delivery of care across six key characteristics of OPC settings. Team composition is routinely reconfigured across professional group boundaries, and is informally formulated ad hoc to best address the issues faced by presenting patients. While we observed a degree of fluidity in team membership, this represents one of many possible models of team care that have been described [1–4]. As observed in other hospital settings [44], features of the design and layout played a role in shaping OPCs, including co-location of departments and professionals. Purpose-designed standalone treatment spaces may increase efficiency and safety, but reduce the opportunity for incidental inter-departmental interactions which provide opportunities for communication about patients and may provide a foundation for care-enhancing social networks; such spaces may need to create artificial mechanisms for building collaborative relationships. Similarly, cluttered treatment spaces need to ensure opportunities for clinicians to focus during their patient interactions, and complex tasks. Further investigation of the design and physical layout of treatment areas is important as recent evidence suggests these may influence the proximity between clinicians, as well as their ability to observe patients undertaking chemotherapy, and thus has patient safety implications [45]. Communication within and between services was a widely-recognised challenge, with staff adopting a range of strategies to enhance responsiveness and mitigate the risk of any possible negative impacts on patients.

Complexity is an underpinning characteristic of OPC care. Care delivery relies on interaction between networks of semi-autonomous agents, for whom misalignment of priorities can pose a barrier to collaboration; time-pressures appear to be a major source of this misalignment. Self-organisation capability, the exercise of judgement and the presence of bridges across networks, facilitate care delivery and enable the coordination of activities. Culture is also a fundamental characteristic of MDC. Here, respect and understanding among professionals, the cultural transmission of institutional expertise and social conventions, as well as the sustaining of inter-professional relationships, served as enablers. From an economic perspective, future research could harness understanding of differences in organisational cultures and any strategies used to develop this culture, between sites, to identify possible avenues for investment. The necessity to go “above and beyond” was commonly observed in all settings, reflecting resource constraint or the frequency of non-standard presentations. Further, the capacity needed to support MDC delivery was understood to encompass the requisite staffing to enable ad hoc and patient-responsive care in the OPC.

Care coordination has been previously highlighted as challenging to achieve across the patient journey, and this study extends the understanding of coordination in ambulatory settings [20, 21, 40, 46]. Issues raised elsewhere, such as the potential for the receipt of conflicting information and poor information flow, were also relevant in these settings [20, 23, 24]. Professionals in the study were acutely aware of potential pitfalls (e.g., double-checking the accuracy of information) and formed proactive strategies, including extending their capacity, to mitigate these risks. This is potentially exacerbated by the knowledge that immediate workload pressures can lead to delayed entry of information into electronic record systems, or for that information to be unclear. Staff members were observed to use all the resources at their disposal in this regard and were often guided by an in-depth knowledge of the institution and their colleagues. On the one hand, there may be opportunities here to identify the key pieces of information, and ensure that it is easy for staff to enter this key information into electronic systems record in real time, to increase efficiency. On the other, there is clear risk that the observed model is unsustainable as demand increases, unless matched by an equivalent increase in staff.

Professionals in the research not only sought to shield patients from disruptions to the continuity of their care but, as is congruent with best practice [5], to also provide whole-of-person care. Ambulatory settings are hubs which draw together networks of professionals and offer a critical touchpoint for identifying unmet patient needs. This setting may be especially important for patients with cancer, given the range of medical and non-medical issues impacting them [6–9].

Nursing scholarship suggests that time and workload pressure can be a barrier to the provision of the relational aspects of care, including the detection of psychosocial, educational and medical needs, and in such circumstances, more visible, physical or task-oriented aspects of care are privileged [9, 47]. The concerns of staff members about managing workloads and the fear expressed of letting team members or patients down, is a well-cited challenge for nurses [48, 49]. We found that staff members adopted a range of prioritisation and multi-tasking strategies to help manage this work. This included strategies documented elsewhere [9, 47], such as embedding aspects of relational care into other tasks, for example, much of the discussion about wellbeing occurred while the nurse was preparing the patient to receive chemotherapy. These pressures, of course were observed to impact other professional groups working in the clinics too, and it is noted that there is considerable variability in staff patient ratios for outpatient cancer care [50–54].

**Fig. 2:**
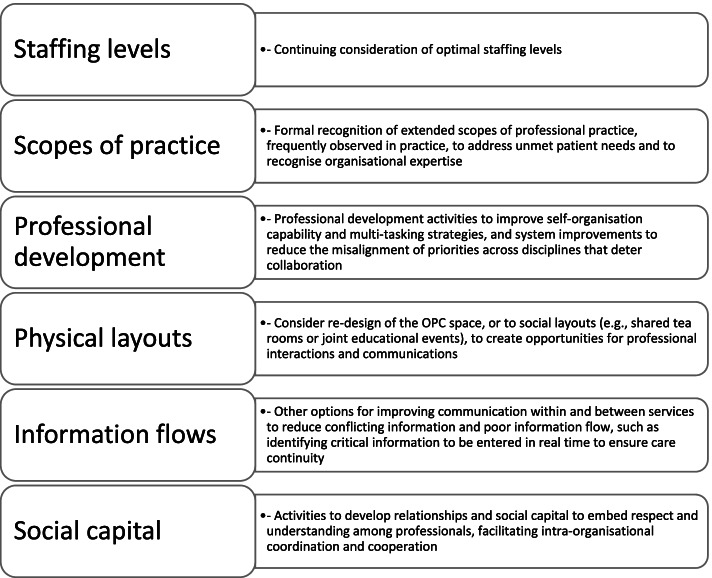
Suggested actions to advance the delivery of cancer services in OPCs

From an improvement perspective, the findings from this paper suggest potential actions to advance the delivery of cancer services in OPCs. Figure 2 summarises actions suggested by this study in areas including staffing numbers, scopes of practice and professional development, reconsidering physical layouts to enhance communication opportunities, identifying critical information flows, and creating opportunities to build relationships within and between teams.

Individual OPCs should consider the expected value of the above and other actions in their local context. Continued research on the costs and effects of actions to improve the safety and quality of outpatient clinic flows is also required.

### Limitations

The study had limitations. The key informant sample comprised professionals from cancer nursing or care coordination backgrounds. While this was informative for learning about point of care processes for nurses, the perspectives of other professional groups involved in this care would have been beneficial. It was not possible to observe closed door consultations or consultations with doctors, which meant that the majority of the observations concerned outpatient waiting areas for radiation therapy and chemotherapy and chemotherapy treatment areas. Other relevant, and potentially connected, observations may have been missed.

Moreover, some of the participating hospitals have cancer services separate from these generic treatment areas and these were not studied; for example, a small number of services are delivered through multidisciplinary clinics where a patient comes in to sequentially consult with multiple clinicians and perhaps participate in the MDTM. While this limits what could be observed, this was necessary to ensure patient privacy and confidentiality, and to minimise the burden of research on clinics. Further, it was not feasible incorporate the study of electronic health records in this ethnographic investigation which limits our analysis, given the importance of the modes of interaction in the course of practice and their implications for how staff manage their time. Observations concerning care for patients for whom English language proficiency is a barrier suggests that additional considerations and support are needed (e.g., the involvement of interpreters) in MDC delivery, and, as such, merits dedicated study. We also noted the way in which patient safety-promoting behaviours and interactions were threaded throughout the observational data collected; we did not include this as a specific interview prompt, which may have influenced the way themes emerged. Future research efforts could incorporate an explicit focus on behaviours and interactions which promote safety in these settings. Qualitative research does not seek to provide generalisations or quantifiable answers [36, 37], and findings need to be interpreted relative to these contexts.

## Conclusion

Application of multiple lenses through which to see the study, namely complexity, coordination, culture and capacity, prompts engagement beyond work-as-imagined to appreciate the processes and resources which support work-as-done. The value of social ties in helping to bridge networks and the employment of social capital to sustain performance, even under pressure, underscores the importance of attending to culture in the study of MDC. Moreover, it speaks to the need for health services to invest in building and supporting and coordinating these critical relationships. The precise nature of the social coordination, built on connections, including bridges across networks, represents as multiple social network analyses, whereby those who create the bridges are ‘boundary spanners’ [55].

## Data Availability

The datasets generated and analysed during the current study are not publicly available due to the conditions of ethical approval but are available from the corresponding author on reasonable request.

## Abbreviations

MDC: Multidisciplinary care
OPC: Outpatient clinic
MDTM: Multidisciplinary team meeting
